# A dataset of 112 ligands for the preconcentration of mercury, uranium, lanthanum and other pollutants and heavy metals in water

**DOI:** 10.1016/j.dib.2020.105236

**Published:** 2020-02-04

**Authors:** N. Kallithrakas-Kontos, P. Boultadaki, S. Foteinis

**Affiliations:** a Technical University of Crete, Laboratory of Analytical and Environmental Chemistry, University Campus, GR-73100 Chania, Greece; b Public Power Corporation (PPC) Renewables S.A., GR-153 43, Attica, Greece

**Keywords:** Analytical chemistry, Water pollution, Environmental monitoring and assessment, Heavy metals, EDXRF, Ligand, Preconcentration

## Abstract

This dataset manuscript describes the preparation procedure and lists the preconcentration efficiency of 112 ligands, immobilized on solid-state polymer membranes, for pollutants/elements monitoring in tap water and in environmentally relevant water matrices. Specifically, the energy dispersive X-ray fluorescence (EDXRF) spectra are presented, along with the preconcentration efficiency of each ligand in tap water. The main materials required for membrane preparation include the membrane matrix, a plasticizer, an ionophore, a catalyst (used only when producing anion-selective membranes), and a complexing agent, i.e. ligand. These are simply mixed, applied on a desired surface, here on a BoPET (biaxially-oriented polyethylene terephthalate) film (Mylar®), and left to dry and solidify, producing anion- or cation-selective membranes. Once the membranes are produced, they can be used even by non-specialised personnel directly on the field, which could be of particular importance for low and middle income countries (LMIC) and for remote or insular areas. The membranes can be functionalised with different ligands, suggesting that they can be used for identifying a vast array of different pollutants/elements in water matrices. Here a dataset of 112 ligands, immobilized on anion-selective membranes, are presented in terms of calcium (Ca), iron (Fe), nickel (Ni), zinc (Zn), antimony (Sb), lanthanum (La), uranium (U), copper (Cu), and gold (Au) preconcentration in tap water. Strontium (Sr) was also attempted to be measured, however, quantifiable results were not obtained. Furthermore, data for mercury (Hg) preconcentration, in cation-selective membranes, are also given. The enclosed data show that the most promising ligand for Hg, Ca, Fe, Ni, Zn, Sr, La, U, Cu, and Au preconcentration were 4-(2-Pyridylazo)resorcinol, Eriochrome Black T, di-Ammonium hydrogen citrate, 1,5-Diphenylcarbazide, dithizone, 1,1'-Carbonyldiimidazole, Bis(cyclopentadienyl)titanium dichloride, sodium dibenzyldithiocarbamate, calconcarbonsaure, and dibenzoylmethane, respectively. Interpretation of the data can be found in our previous work [1]. Overall, the main intention of this dataset manuscript is to communicate and promote the adoption of the proposed method by researchers and the water industry alike. This could further advance the method and encourage the assessment of additional ligands or/and pollutants/elements, including heavy metals which are typically found in water.

**Table:** Specifications Table

Subject	Analytical Chemistry, Environmental Chemistry, Environmental Engineering
Specific subject area	Environmental monitoring and assessment of water
Type of data	Table: The preconcentration efficiencies, in counts/300s, for 112 ligands and for the Ca, Fe, Ni, Zn, Sr, La, U, Cu, and Au Image: Photographs of the membrane preparations steps. Graph:The tens most promising ligands for Hg, Ca, Fe, Ni, Zn, Sr, La, U, Cu, and Au preconcentration, in counts/300s Figure: The EDXRF spectra for Hg preconcentration in tap water using i) 4-(2-Pyridylazo)resorcinol (PAR), ii) thiourea, iii) dithizone, and iv) calconcarbonsaure (CCS) functionalised membranes.
How data were acquired	The data were acquired by Energy dispersive X-ray fluorescence (EDXRF) spectrometry Instruments: Pictures were taken using a compact digital camera. The spectra were obtained by an EDXRF spectrometer Model AMETEK SPECTRO XEPOS unit The spectra were processed with X-Lab Pro 4.0 software, using the TurboQuant screening method.
Data format	The raw *EDXRF spectra in .txt format, individually presented and including the experimental conditions* The processed spectra in shown in*, Tables in .docx, diagrams in .xlxs format* *The images of the membrane preparations steps in. jpg format*
Parameters for data collection	The raw EDXRF spectra were collected using the secondary/molybdenum mode at 40 kV and 0.9 mA, with helium gas flushing, and 300 s irradiation duration.
Description of data collection	Pictures were taken at the laboratory showing the membrane preparation procedure. The EDXRF spectra were collected by an AMETEK SPECTRO XEPOS unit, using the secondary/molybdenum mode at 40 kV and 0.9 mA, with helium gas flushing, and 300 s irradiation duration
Data source location	Laboratory of Analytical and Environmental Chemistry/Technical University of Crete/University Campus/Chania/Greece
Data accessibility	With the article
Related research article	N. Kallithrakas-Kontos, S. Foteinis, E. M. Vazgiouraki, A. G. Karydas, J. Osán, E. Chatzisymeon, Solid-state polymer membranes for simple, sensitive, and low-cost monitoring of mercury in water, Science of The Total Environment, 697, 2019, 134099, https://doi.org/10.1016/j.scitotenv.2019.134099

**Table:** 

**Value of the Data** • The dataset regarding the membrane preparation procedure, i.e. the enclosed pictures and description, can be put forward by other researchers and the water industry alike to test, further improve, and apply the method to address real world problems. The membranes are easily reproducible, cost-effective, and easy to use even by non-specialised personnel. Therefore, the dataset regarding the membrane preparation procedure also can encourage the application of the method in low and middle income countries (LMIC), where the identification and monitoring of clean water resources is a matter of emerging concern [2]. • More importantly, the raw and processed EDXRF datasets covering 112 ligands, which were screened in terms of targeted pollutants/elements identification and quantification in tap water, can provide context with the literature and promote further research to furnish the proposed method, both in terms of efficiency and practicality. • Finally, the data can be put forward by other researchers to examine additional ligands and/or pollutants/elements, thus complement the enclosed dataset.

## 1 Data description

**Fig. 1:**
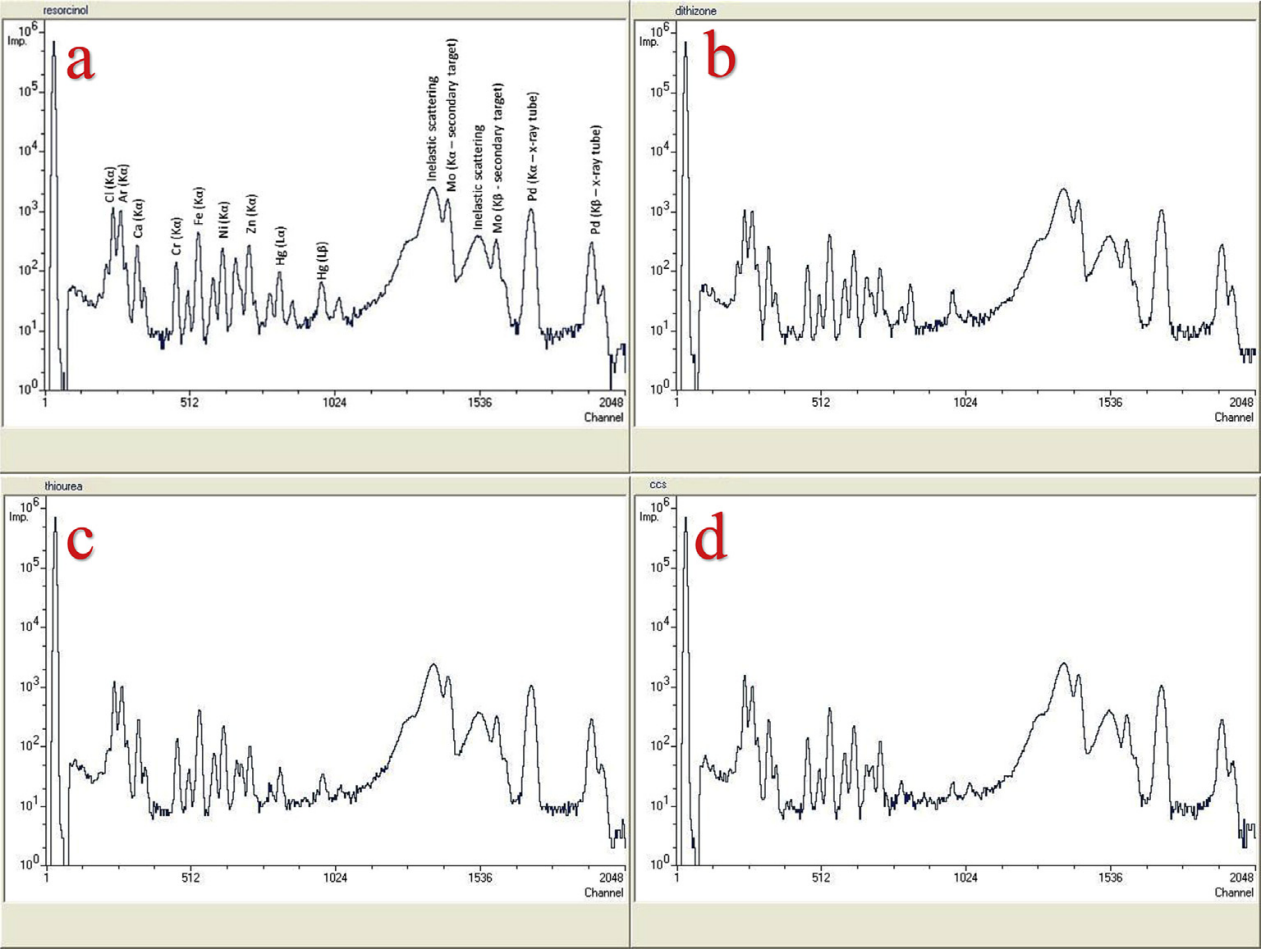
The preconcentration efficiency for Hg(II) in tap water and for four different membranes functionalised with a) resorcinol (PAR), b) dithizone, c) thiourea, and d) CCS. Around 12.643 channels correspond to 1 keV.

**Fig. 2:**
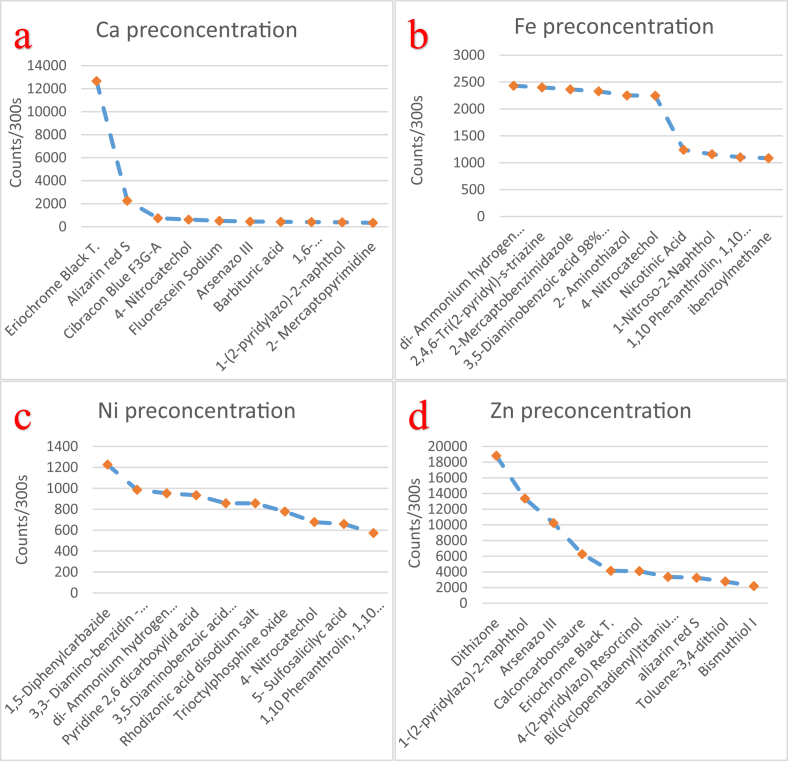
Graphic presentation of the data for the ten most promising ligands, from higher to lower score, for a) Ca, b) Fe, c) Ni, and d) Zn preconcentration in tap water.

**Fig. 3:**
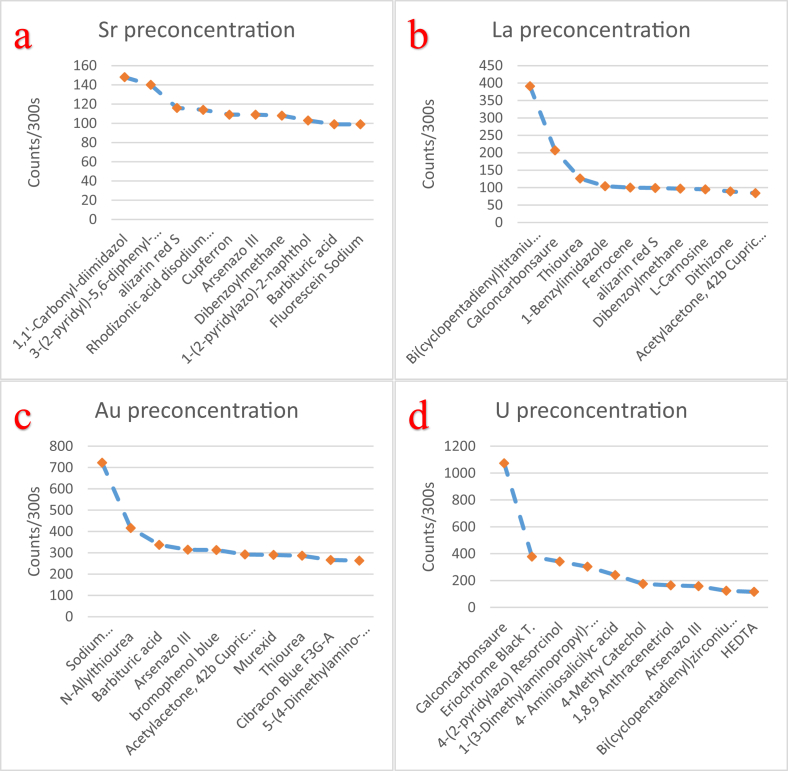
Graphic presentation of the data for the ten most promising ligands, from higher to lower score, for a) Sr, b) La, c) Au, and d) U preconcentration in tap water.

**Fig. 4:**
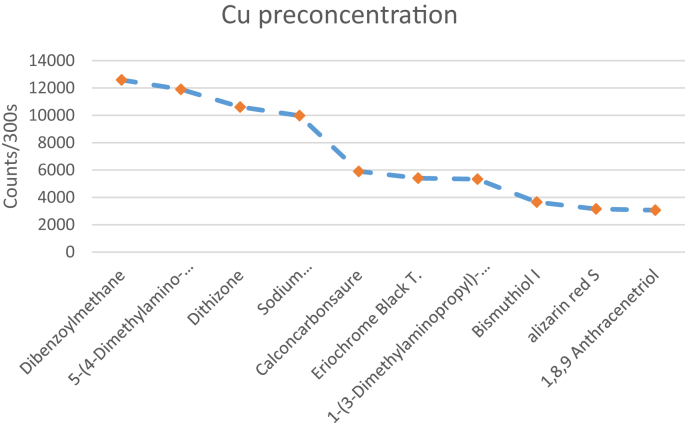
Graphic presentation of the data for the ten most promising ligands, from higher to lower score, for Cu preconcentration in tap water.

**Fig. 5:**
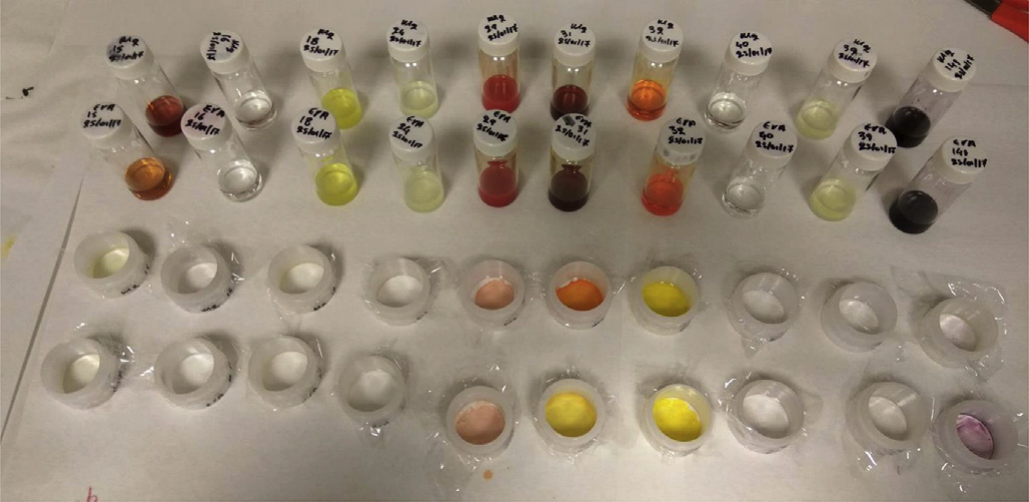
A few of the prepared membranes in liquid form and their application in XRF sample cups.

**Fig. 6:**
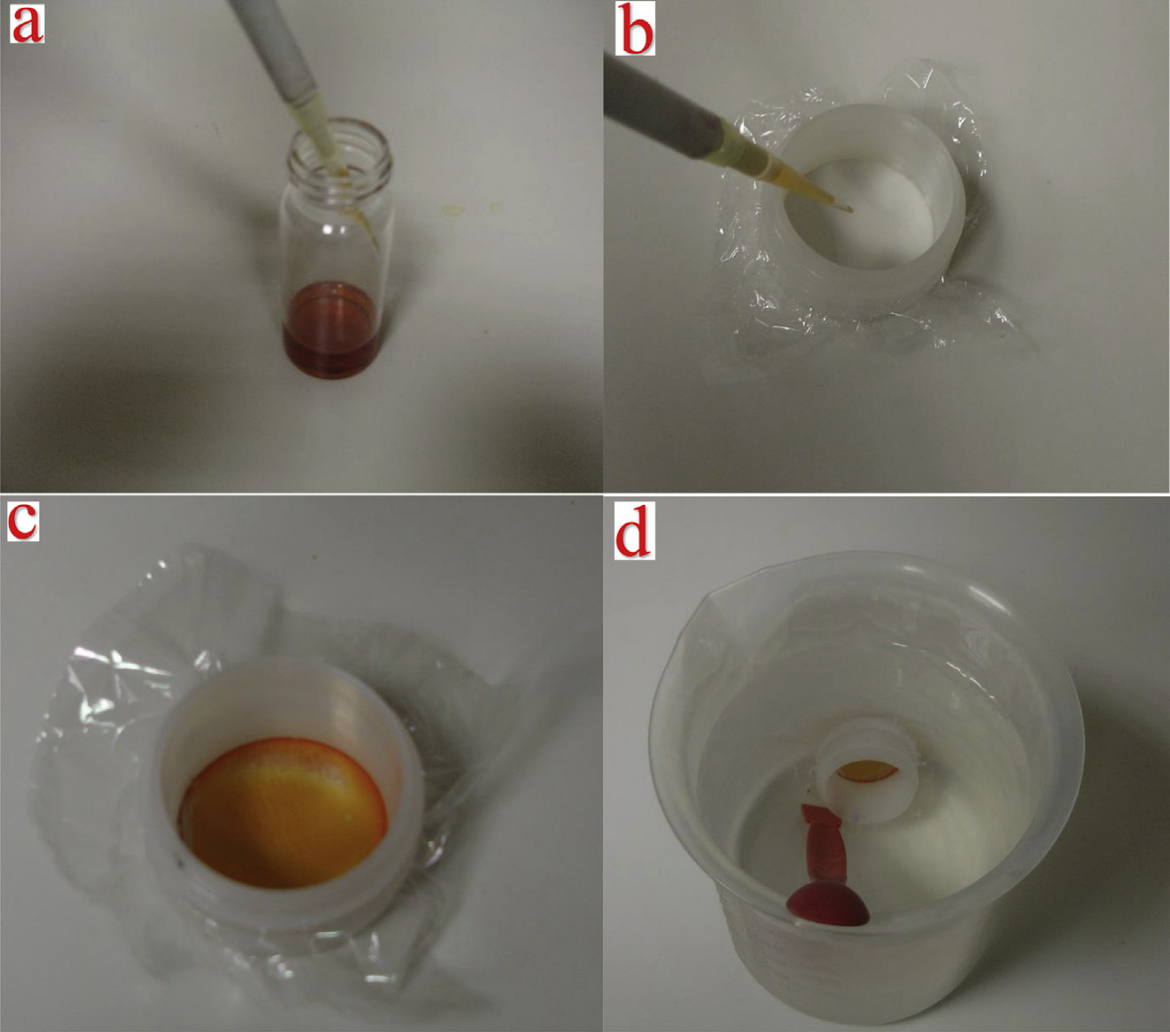
a) the membrane solution, b) the double open ended XRF sample cup along with the Mylar® film, c) the solidified membrane, and d) the immersed membrane in the water matrix.

**Fig. 7:**
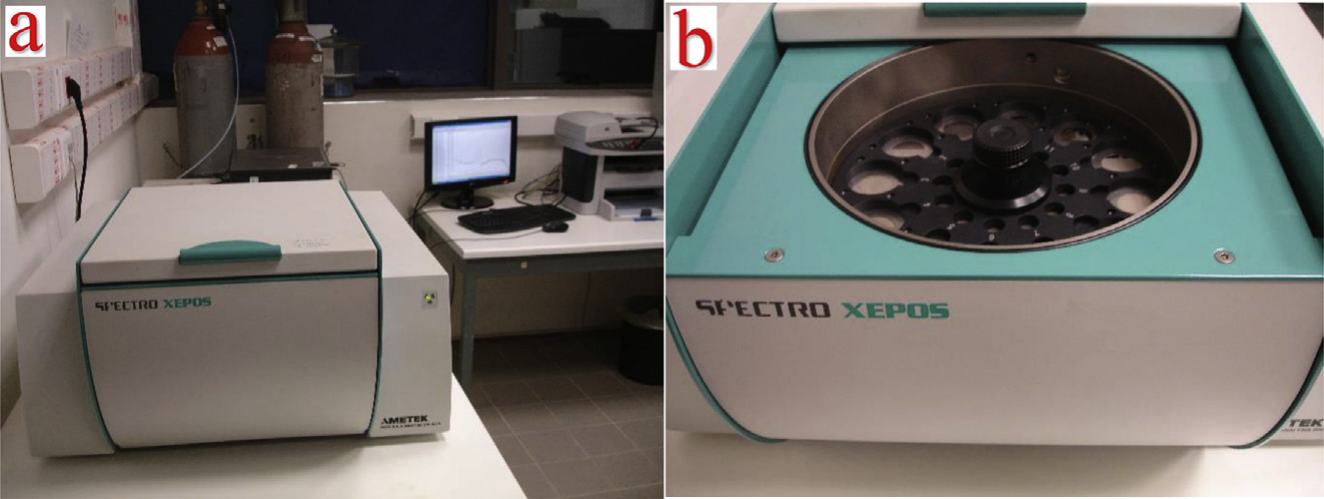
a) the AMETEK SPECTRO XEPOS unit used for the EDXRF measurements, and b) the 12 position autosampler.

The effectiveness of solid-state polymer membranes for mercury preconcentration in water was examined in a recent work of our group [1]. In this data article the EDXRF spectra (Fig. 1) along with the raw EDXRF data for Hg preconcentration in water are given. In addition, the raw EDXRF data regarding the screening of 112 ligands, immobilized on anion-selective solid-state membranes, for calcium (Ca), iron (Fe), nickel (Ni), zinc (Zn), strontium (Sr), lanthanum (La), uranium (U), copper (Cu), and gold (Au) preconcentration in tap water are enclosed (multimedia component 1-112). Table 1 list the quantitative results for the 112 ligands, while the ten most promising ligands for the preconcentration of the targeted elements is shown in Fig. 2, Fig. 3, Fig. 4 and listed in Table 2, Table 3, Table 4, Table 5, Table 6, Table 7, Table 8, Table 9, Table 10. The quantitative data used to generate Fig. 2, Fig. 3, Fig. 4 are also enclosed (Fig. 1, Fig. 2, Fig. 3.xlxs). To provide context, the blank spectrum of the Mylar® film alone (Mylar blank), as well as the spectrum of the membrane before being immersed in the water matrix (EVA blank) are also given in the enclosed dataset. Finally, in Fig. 5, Fig. 6, Fig. 7 photographs of experimental procedure and instrumentation, which has been previously described [1], are shown.

**Table 1:** The examined 112 different ligands, immobilized on anion-selective membranes, along with the corresponding efficiency for Ca, Fe, Ni, Zn, Sr, La, U, Cu, and Au preconcentration in tap water and in counts/300 s.

Ligand		Ca	Fe	Ni	Zn	Sr	Au	U	La	Cu
**Counts/300 s**										
1. Amarillo de titan (Titan yellow)		14	324	0	42	0	119	31	40	127
2. Methyl orange 0.1%		32	385	118	111	69	136	40	70	91
3. Alizarin Red S		2259	156	100	3246	116	128	0	99	3153
4. Bromophenol blue		0	242	0	0	0	313	0	0	0
5. Azul blue de bromothymol 0.04%		0	187	104	0	0	43	0	0	0
6. Bromocresol green		0	95	0	50	0	133	0	0	77
7. Eriochrome cyanine R		0	4	0	873	0	90	0	0	2163
8. Hydroxynaphthol blue		0	0	0	271	0	64	0	0	1803
9. Bromothymol blue		6	0	94	0	117	135	0	44	0
10. Eriochrome Black T		12662	235	0	4132	0	175	378	78	5402
11. 1-(2-Pyridylazo)-2-naphthol		381	289	265	13357	103	217	0	0	1713
12. 1-(3-Dimethylaminopropyl)-3-ethylcarbodiimide, polymer-bound		78	139	263	0	0	126	303	0	5333
13. 1-Butyl-3-methylimidazolium hexafluorophosphate		61	831	344	362	0	86	0	0	188
14. 1,10-Phenanthroline monohydrate		0	1102	572	317	0	10	0	0	185
15. D-(-)-Fructose		109	299	163	52	0	52	0	26	12
16. 1,10-Phenanthroline 1/40 M Ferroin solution		0	222	211	0	0	159	0	0	35
17. 1,1'-Carbonyldiimidazole		93	176	100	269	148	98	0	0	97
18. 1,5-Diphenylcarbazide		31	170	1226	0	0	117	0	0	7
19. 1,6-Diaminohexane-N,N,N′,N′-tetraacetic acid		404	356	160	0	35	22	0	42	0
20. 1-Benzylimidazole		132	162	145	0	64	108	44	104	19
21. 1-Butyl-3-methylimidazolium tetrafluoroborate		53	675	38	283	0	139	0	35	271
22. 1-Hexanesulfonic acid sodium salt		29	750	403	0	0	100	0	65	0
23. 1-Nitroso-2-naphthol		159	1157	103	879	0	61	0	26	776
24. 2-Aminobenzothiazole		0	508	36	287	0	45	0	64	143
25. 2-Aminothiazole		0	2250	402	119	0	161	0	0	167
26. 2-Mercaptopyrimidine		338	942	41	702	81	256	0	34	825
27. 2-(5-Bromo-2-pyridylazo)-5-(diethylamino)phenol		0	111	0	17	0	141	0	36	156
28. 2,4,6-Tris(2-pyridyl)-s-triazine		86	371	90	172	0	88	0	20	39
29. 2-Hydroxybiphenyl 98%		11	655	461	100	0	154	0	35	100
30. 2-Mercaptobenzimidazole		0	64	0	1759	0	67	0	35	1391
31. 2-Mercaptobenzothiazole		0	426	293	246	0	96	0	0	593
32. 3-(2-Pyridyl)-5,6-diphenyl-1,2,4-triazine-p,p′-disulfonic acid		169	515	40	703	140	0	0	29	242
33. 3,3′-Diaminobenzidine tetrahydrochloride hydrate		0	2364	986	0	0	230	0	43	661
34. 3,5-Diaminobenzoic acid 98%		4	2328	857	117	53	65	0	25	498
35. 4- Aminosalicylic acid		0	13	107	0	0	218	241	36	511
36. 4-(4-Nitrophenylazo)-1-naphthol		0	258	77	161	0	123	0	27	101
37. 4- Nitrocatechol		622	2246	677	311	0	210	0	37	1949
38. 4-(2-Pyridylazo)resorcinol		22	498	364	4085	0	210	341	0	439
39. 4-(2-Thiazolylazo)resorcinol		148	950	99	491	0	26	110	0	199
40. 4-Chlorophenol		236	648	113	645	0	105	0	70	220
41. 4-Chlorophenyl sulfoxide		195	524	47	0	0	218	0	24	40
42. 5-Amino-1,3,4-thiadiazole-2-thiol		0	284	150	64	0	236	0	58	763
43. 5-Sulfosalicylic acid		43	2401	659	17	0	81	0	44	0
44. 5-(4-Dimethylaminobenzylidene)-rhodanine		164	568	392	323	0	263	0	32	11903
45. 3-(2-Pyridyl)-5,6-diphenyl-1,2,4-triazine		0	532	152	163	86	50	0	40	32
46. 8-Hydroxyquinoline		259	307	310	131	0	0	0	50	179
47. a-Benzoin oxime		71	492	188	17	0	62	0	56	1483
48. Cupric acetylacetonate		227	533	0	335	0	292	0	84	565
49. Ammonium hexacyanoferrate(II) hydrate		289	12039	0	833	0	91	0	36	254
50. Ammonium pyrrolidinedithiocarbamate		63	187	88	228	0	31	0	0	250
51. Antipyrine 98%		7	49	49	98	0	81	0	31	255
52. Barbituric acid		430	676	189	332	99	337	0	66	305
53. Bis(cyclopentadienyl)titanium dichloride		90	467	267	3353	0	100	0	391	2550
54. Bis(cyclopentadienyl)zirconium dichloride		297	588	171	263	0	164	124	66	515
55. Bismuthiol I		1	31	0	2182	0	185	0	0	3656
56. N,N-Diethyl-p-phenylenediamine sulfate salt		0	0	0	0	0	55	0	35	0
57. Calconcarbonsaure		251	463	351	6267	30	224	1073	207	5899
58. Cibacron Blue F3G-A		739	840	258	255	65	266	0	35	622
59. Cytidine, cell culture		292	315	0	240	0	136	0	37	147
60. di-Ammonium hydrogen citrate		158	2431	952	90	0	174	67	80	231
61. Dibenzoylmethane		309	1085	219	658	108	211	66	97	12590
62. Dimethylglyoxime		0	428	310	34	0	40	0	27	10
63. 4-Methylcatechol		0	163	38	1059	0	108	175	0	1340
64. Diphenylcarbazone		136	366	388	9	0	169	0	33	6
65. Dithiooxamide		0	232	198	654	0	86	0	70	899
66. Dithizone		264	346	270	18809	0	192	46	89	10609
67. Epichlorohydrin		0	693	465	203	0	91	0	0	59
68. Fluorescein sodium		509	563	173	268	99	162	0	53	472
69. Gluconic acid - Potassium salt		0	199	97	0	0	109	75	46	12
70. HEDTA		135	443	290	374	0	167	116	63	109
71. Hippuric acid 98%		0	1006	63	12	88	51	0	36	40
72. Hydrazine sulfate		0	248	383	5	0	121	0	0	0
73. Mercury ionophore I		197	0	0	0	0	91	0	38	0
74. Michler's Ketone		0	19	375	0	0	65	0	35	25
75. Murexide		0	153	108	401	0	290	0	35	763
76. N-Benzoyl-N-phenylhydroxylamine		0	340	138	70	0	198	0	57	53
77. N,N,N',N'-Tetramethyl-1,8-naphthalenediamine		0	130	83	20	0	152	0	41	20
78. N-Hydroxysulfosuccinimide sodium salt		0	554	135	124	0	173	0	0	141
79. Nicotinic acid		194	1240	107	167	90	208	0	38	146
80. Nitroso-R-salt		0	298	0	0	0	106	0	39	257
81. o-Dianisidine		0	132	29	27	62	159	43	35	159
82. Orotic acid		99	150	126	245	0	85	0	48	138
83. Sodium oxalate		0	184	0	104	95	181	70	44	93
84. Phenyl acetate 99%		7	572	128	161	0	99	0	66	84
85. 2,6-Pyridinedicarboxylic acid		92	0	934	0	0	48	0	34	
86. Quinaldic acid 98%		248	278	391	0	0	0	0	30	0
87. Rhodizonic acid disodium salt		40	558	857	706	114	108	0	46	608
88. Sodium cyanide		53	376	43	0	0	120	0	63	13
89. Sodium dibenzyldithiocarbamate		32	661	149	1291	0	722	0	33	9976
90. Sodium diethyldithiocarbamate trihydrate		34	430	105	106	0	56	82	45	497
91. syn-2-Pyridinealdoxime		39	266	98	44	0	93	0	57	23
92. Thymine		0	416	0	80	88	85	0	0	0
93. Titriplex II (ethylenedinitrilotetraacetic acid)		25	0	0	0	80	47	47	62	0
94. Triethylenetetramine-N,N,N′,N′′,N′′′,N′′′-hexaacetic acid		45	431	654	0	64	13	0	42	0
95. Trioctylphosphine oxide		0	179	778	0	0	191	62	34	50
96. Xylenol orange, sodium salt		0	337	226	32	69	83	52	25	0
97. Ν,Ν,Ν',Ν' -Tetraacetic acid		0	78	0	0	0	39	54	43	0
98. Ν-Allylthiourea		0	395	0	143	0	416	107	41	403
99. Menthol		77	405	227	110	0	62	0	25	59
100. Cupferron		7	650	260	126	109	119	0	25	173
101. Thiourea		0	307	441	0	0	286	0	126	203
102. Starch		65	310	128	153	0	134	0	32	73
103. Toluene-3,4-dithiol		33	422	50	2774	0	119	0	0	2003
104. 1,1′-Carbonyl-di-(1,2,4-triazole)		32	950	200	23	0	42	0	45	23
105. L-carnosine		163	542	0	245	0	118	0	95	205
106. Uracil		74	353	277	138	0	97	0	66	108
107. 1,8,9-Anthracenetriol		60	74	47	407	0	137	164	45	3063
108. 3,3'-Diaminobenzidine		0	0	0	0	0	0	72	0	1108
109. o-Phenanthroline		0	0	0	0	0	0	84	0	0
110. Citric acid		0	0	15	0	0	76	0	0	0
111. Arsenazo III		446	190	108	10232	109	314	158	62	1391
112. Ferrocene		0	899	59	0	0	210	0	100	98

**Table 2:** The ten most promising ligands for the calcium (Ca) preconcentration in the solid-state membranes.

Ligand	Counts/300s
Eriochrome Black T	12662
Alizarin red S	2259
Cibacron Blue F3G-A	739
4-Nitrocatechol	622
Fluorescein sodium	509
Arsenazo III	446
Barbituric acid	430
1,6-Diaminohexane-N,N,N′,N′-tetraacetic acid	404
1-(2-Pyridylazo)-2-naphthol	381
2-Mercaptopyrimidine	338

**Table 3:** The ten most promising ligands for the iron (Fe) preconcentration in the solid-state membranes.

Ligand	Counts/300s
di-Ammonium hydrogen citrate	2431
2,4,6-Tris(2-pyridyl)-s-triazine	2401
2-Mercaptobenzimidazole	2364
3,5-Diaminobenzoic acid 98%	2328
2-Aminothiazole	2250
4-Nitrocatechol	2246
Nicotinic Acid	1240
1-Nitroso-2-naphthol	1157
1,10-Phenanthroline monohydrate	1102
Dibenzoylmethane	1085

**Table 4:** The ten most promising ligands for the nickel (Ni) preconcentration in the solid-state membranes.

Ligand	Counts/300s
1,5-Diphenylcarbazide	1226
3,3′-Diaminobenzidine tetrahydrochloride hydrate	986
di-Ammonium hydrogen citrate	952
2,6-Pyridinedicarboxylic acid	934
3,5-Diaminobenzoic acid 98%	857
Rhodizonic acid disodium salt	857
Trioctylphosphine oxide	778
4-Nitrocatechol	677
5-Sulfosalicylic acid	659
1,10-Phenanthroline monohydrate	572

**Table 5:** The ten most promising ligands for the zinc (Zn) preconcentration in the solid-state membranes.

Ligand	Counts/300s
Dithizone	18809
1-(2-Pyridylazo)-2-naphthol	13357
Arsenazo III	10232
Calconcarbonsaure	6267
Eriochrome Black T	4132
4-(2-Pyridylazo)resorcinol	4085
Bis(cyclopentadienyl)titanium dichloride	3353
Alizarin red S	3246
Toluene-3,4-dithiol	2774
Bismuthiol I	2182

**Table 6:** The ten most promising ligands for strontium (Sr) preconcentration in the solid-state membranes.

Ligand	Counts/300s
1,1'-Carbonyl-diimidazol	148
3-(2-Pyridyl)-5,6-diphenyl-1,2,4-triazine-p,p′-disulfonic acid	140
Alizarin Red S	116
Rhodizonic acid disodium salt	114
Cupferron	109
Arsenazo III	109
Dibenzoylmethane	108
1-(2-Pyridylazo)-2-naphthol	103
Barbituric acid	99
Fluorescein sodium	99

**Table 7:** The ten most promising ligands for the lanthanum (La) preconcentration in the solid-state membranes.

Ligand	Counts/300s
Bis(cyclopentadienyl)titanium dichloride	391
Calconcarbonsaure	207
Thiourea	126
1-Benzylimidazole	104
Ferrocene	100
Alizarin Red S	99
Dibenzoylmethane	97
L-carnosine	95
Dithizone	89
Cupric acetylacetonate	84

**Table 8:** The ten most promising ligands for the gold (Au) preconcentration in the solid-state membranes.

Ligand	Counts/300s
Sodium dibenzyldithiocarbamate	722
Ν-Allylthiourea	416
Barbituric acid	337
Arsenazo III	314
Bromophenol blue	313
Cupric acetylacetonate	292
Murexide	290
Thiourea	286
Cibacron Blue F3G-A	266
5-(4-Dimethylaminobenzylidene)-rhodanine	263

**Table 9:** The ten most promising ligands for the uranium (U) preconcentration in the solid-state membranes.

Ligand	Counts/300s
Calconcarbonsaure (CCS)	1073
Eriochrome Black T	378
4-(2-Pyridylazo)resorcinol	341
1-(3-Dimethylaminopropyl)-3-ethylcarbodiimide, polymer-bound	303
4- Aminiosalicilyc acid	241
4-Methy Catechol	175
1,8,9-Anthracenetriol	164
Arsenazo III	158
Bis(cyclopentadienyl)zirconium dichloride	124
HEDTA	116

**Table 10:** The ten most promising ligands for the copper (Cu) preconcentration in the solid-state membranes.

Ligand	Counts/300s
Dibenzoylmethane	12590
5-(4-Dimethylaminobenzylidene)-rhodanine	11903
Dithizone	10609
Sodium dibenzyldithiocarbamate	9976
Calconcarbonsaure	5899
Eriochrome Black T	5402
1-(3-Dimethylaminopropyl)-3-ethylcarbodiimide, polymer-bound	5333
Bismuthiol I	3656
Alizarin Red S	3153
1,8,9-Anthracenetriol	3063

Specifically, the data regarding the Hg(II) preconcentration were obtained using cation-selective polymer-based membranes. The EDXRF spectra for the cation-selective membranes, using polyvinyl chloride (PVC) as the membrane matrix, and for four examined ligands, i.e. i) 4-(2-Pyridylazo)resorcinol (PAR), ii) thiourea, iii) dithizone, and iv) calconcarbonsaure (CCS) are shown in Fig. 1. As mentioned above, the raw data of the EDXRF spectra are also enclosed in this dataset manuscript. As shown in Fig. 1, resorcinol (PAR) appears to be the most promising ligand, judging from the Hg peak in the corresponding spectrum (Fig. 1a), for aqueous Hg(II) preconcentration, by and large, followed by dithizone and thiourea. On the other hand, CCS had a very low preconcentration efficiency, suggesting its limited potential for mercury preconcentration in water matrices. However, as will be discussed below, CCS was found particularly promising for U preconcentration in water.

In addition, the membranes were also screened using 112 different ligands were immobilized on the solid-state membranes and were screened regarding their preconcentration efficiency for the determination of 9 different pollutants/elements, i.e. Ca, Fe, Ni, Zn, Sr, La, U, Cu, and Au. The quantitative results of the EDXR measurements, along with the name of each examined ligand, are given in Table 1. It should be noted that the membranes were also screened in terms of antimony (Sb) preconcentration in water, however no quantifiable results were obtained and thus Sb is not included in Table 1. Specifically, during the screening process the efficiency of both anion- and cation-selective membranes was examined and it was identified that the vast majority of the examined pollutants/elements were preferably complexing with the ligand that was immobilized on anion-selective membranes. For this reason the 112 ligands were screened using anion-selective membranes, with the membrane matrix being ethylene vinyl acetate (EVA). To this end, 1 L of tap water was spiked with 20 μg L^−1^ Au, 20 μg L^−1^ La, 50 μg L^−1^ U, 50 μg L^−1^ Sb, and 100 μg L^−1^ Sr. Then, each membrane was immersed in the spiked tap water and left for 24 h to reach equilibrium. In this screening process the water matrix (tap water) was not spiked with Hg(II), since the Hg(II) spectrum could overlap and largely interfere with that of Au, thus making Au quantification difficult. Furthermore, Ca, Fe, Ni, Zn, and Cu are naturally present in tap water and in many instances (i.e. in many of the examined ligands) these elements were preconcentrated on the membranes and thus were able to be quantified, as shown in Table 1. Finally, as mentioned above the raw EDXRF spectra, which include also the non-quantifiable Sb concentrations, are enclosed in this dataset manuscrip. In the context of this work, the quantification of La, U, Au was achieved using the Lα lines, while for Ca, Fe, Ni, Zn, Sr, Sb, and Cu the Kα lines were used. However, as already mentioned Sb did not yield quantifiable results and hence is not included in Table 1.

From Table 1 it is possible to identify the most promising functionalised membranes for each examined element, i.e. the most promising ligands since in practise only the ligand is diversified between membranes. Specifically, in the electronically available Table 2, Table 3, Table 4, Table 5, Table 6, Table 7, Table 8, Table 9, Table 10 the ten most promising membranes/ligands for Ca, Fe, Ni, Zn, Sr, La, U, Cu, and Au preconcentration in tap water, along with the achieved efficiency (in counts per 300 s), are given. Furthermore, in Fig. 2 the ten most promising ligands for Ca, Fe, Ni, and Zn preconcentration are shown, in Fig. 3 the ten most promising ligands for Sr, La, Au, and U, and in Fig. 4 the ten most promising ligands for Cu preconcentration in tap water are shown.

As observed in Fig. 2 the most promising ligand, by and large, for Ca preconcentration is Eriochrome Black T. For Fe preconcentration in water six ligands appear to yield very good scores, with di-Ammonium hydrogen citrate having the higher score, while the best ligands for Ni and Zn preconcentration are1,5-Diphenylcarbazide and dithizone, respectively. From Fig. 3 it can be inferred that for Sr preconcentration 1,1'-Carbonyldiimidazole is the most promising ligand, closely followed by 3-(2-Pyridyl)-5,6-diphenyl-1,2,4-triazine-p,p′-disulfonic acid. For La and Au preconcentration in water the most promising ligands are bis(cyclopentadienyl)titanium dichloride and sodium dibenzyldithiocarbamate, respectively, while for U the most promising ligand is, by and large, Calconcarbonsaure (CSS). Finally, from Fig. 4 it is inferred that dibenzoylmethane, closely followed by 5-(4-Dimethylaminobenzylidene)rhodanine, are the most promising ligands for Cu preconcentration in tap water, while dithizone and sodium dibenzyldithiocarbamate were also found promising.

## 2 Experimental design, materials and methods

A main strength of the solid-state polymer membranes lies in the fact that they are fairly simple to produced and used, as is described below. Specifically, in order to produce the membranes, a solution containing the following reagents needs to be prepared. First a polymer, such as EVA or PVC, is used as the membrane matrix. This will be mixed with a plasticizer, here dibutyl phthalate (DBP); an ionophore, here DTNB (5,5′-Dithiobis-(2-nitrobenzoic acid), popularly known as Ellman's reagent); a catalyst, which is only used when producing anion-selective membranes (here the Aliquat® 336 was used); and finally a complexing agent, which is the ligand that was used to functionalise each membrane. The abovementioned chemical reagents are in solid form. For this reasons they were added into small cylindrical bottles, diluted with tetrahydrofuran (THF) and simply shaken for homogenisation (Fig. 5). The reagents concentration for the anion-selective membranes, which were used for the screening of the 112 ligands, was 9.4 g THF, 0.081 g EVA, 0.054 g Aliquat® 336, 0.02 g DTNB, 0.094 g DBP, and 0.015 g ligand. If a cation-selective membrane needs to be prepared then the catalyst, i.e. Aliquat® 336, should be omitted, i.e. not added to the abovementioned mixture. If the membranes are expected to be produced on a more comprehensive scale, mixing could be achieved using more elaborate techniques.

Once the membrane solution is homogenised, through shaking or mixing, this is simply applied on the desired surface, in this case a 2.5 μm thickness Mylar® film that is firmly place in a 32 mm double open ended XRF sample cup. In this work, this was achieved by placing 10 μL of the membrane solution, using a single-channel pipette, directly on the center of the Mylar® film. A spot is created, which was then slowly spread uniformly across the film surface using the pipette tip. Emphasis was given to ensure that the liquid form of the membrane will be spread uniformly on the Mylar® surface, and, to the extent possible, without touching the plastic edges of the XRF sample cups (Fig. 6 a-c). The reason is that the part that is attached to the plastic edges of the sample cup will not be quantified during the EDXRF analysis. Given the large number of membranes that were examined in this work, in general, the liquid form of each membranes was spread relatively uniformly covering all of the Mylar® film surface, while a miniscule amount could be also deposited at the plastic edges of the XRF cups. However, this does not affected the analysis, since this is a comparative study and the same procedure was followed in all the examined membranes. A higher amount of the membrane could be also applied to the Mylar® film, which could make the uniformly application of the liquid form of the membrane easier. Finally, for solvent evaporation and membrane solidification the applied membrane solution is left to dry at room temperature for 24 h (an IR lamp can be used to reduce the drying duration). It should be noted that if the total reflection X-ray fluorescence (TXRF) technique is planned to be used, instead of the EDXRF technique, the membrane solution can be directly applied on the center of the quartz reflector, instead of the Mylar® film, and then left to solidified.

The solidified membrane is then ready to be used. In the context of this work, the prepared membranes were immersed in 1 L of tap water spiked with 20 μg L-1 Au, 20 μg L-1 La, 50 μg L-1 U, 50 μg L-1 Sb, and 100 μg L-1 Sr, as mentioned above. The membranes were left to rest for 24 h inside the water matrix, in order for the pollutants/elements contained in the water matrix to reach equilibrium on the membrane surface (Fig. 6 d). The water matrix can be kept under continuous stirring, which enhances ion mobility and binding on the membrane surface thus lowering detection limits, or left unstirred. Here the water matrix was left unstirred. The reason is twofold. First, the main objective of this study was to compare the different membranes/ligands in terms of pollutants/elements preconcentration efficiency and not identify the detection limits of each examine ligand. Second, the unstirred water matrix requires a simpler configuration, compared to continuous stirring, and is also easier to be use directly on the field and even by non-specialised personnel.

Finally, after 24 h of equilibrium inside the water matrix, the membrane is retrieved, washed with ultrapure water, and left to dry, before being measured by means of an EDXRF unit. Here, the membranes were assessed by an AMETEK SPECTRO XEPOS unit (Fig. 7 a), using the secondary/molybdenum mode at 40 kV and 0.9 mA, with helium gas flushing and 300 s irradiation duration. The unit is equipped with a 12 position autosampler (Fig. 7 b), equipt with trays for different sample diameters (here the 32mm diameter was used). This allows for multiple samples, up to 12, to be measured. The spectra were then processed and quantified by means of the X-Lab Pro 4.0 software and using the TurboQuant method. The above suggest that the proposed method can provide robust results using relatively low irradiation times (i.e. 300 s). Furthermore, due to its simplicity and ability to be applied in unstirred water matrices, this method could be promising for the application of the method in low and middle income countries (LMIC), where the identification and monitoring of fresh water resources is a matter of emerging concern. The method could also achieve very low detection limits, even lower than μg·L^−1^ by means of EDXRF, as was highlighted in our previous work [1]. Overall, the presented data suggests that the proposed solid-state membranes can be a promising method for pollutants monitoring and assessment in water matrices of environmental concern. Furthermore, future works of our group will focus on identifying the sensitivity and the detection limit of the most promising membranes/ligands for each of the examined element.

## Appendix A Supplementary data

Multimedia component 1_amarillo de titan Multimedia component 1

Multimedia component 2_methylorange 0.1_spl_ Multimedia component 2

Multimedia component 3_alizarin red S Multimedia component 3

Multimedia component 4_bromophenol blue Multimedia component 4

Multimedia component 5_azul blue de bromothymol 0.04_spl_ Multimedia component 5

Multimedia component 6_bromocresol green Multimedia component 6

Multimedia component 7_eriochrome cyanine R Multimedia component 7

Multimedia component 8_hydroxynaphthol blue Multimedia component 8

Multimedia component 9_bromothymol blue Multimedia component 9

Multimedia component 10_Eriochrome Black T Multimedia component 10

Multimedia component 11_1-_spl_2-pyridylazo_spl_-2-naphthol Multimedia component 11

Multimedia component 12_1-_spl_3-Dimethylaminopropyl_spl_-3-ethyl carbodiimide polymer bound Multimedia component 12

Multimedia component 13_1_spl_ Butyl-3-methylimidazolium hexafluoro phosphate Multimedia component 13

Multimedia component 14_1_spl_10 Phenanthrolin_spl_ 1_spl_10 Phenanthrolin monohydrat Multimedia component 14

Multimedia component 15_D-Fructose Multimedia component 15

Multimedia component 16_1_spl_10-Phenanthrolin 140 M Ferroin solution Multimedia component 16

Multimedia component 17_1_spl_1_spl_-Carbonyl-diimidazol Multimedia component 17

Multimedia component 18_1_spl_5-Diphenylcarbazide Multimedia component 18

Multimedia component 19_1_spl_6- Diaminohexane_spl_N_spl_N_spl_N_spl__spl_N_spl_ tetraacetic acid Multimedia component 19

Multimedia component 20_1-Benzylimidazole Multimedia component 20

Multimedia component 21_1-Butyl-3-methylimidazolium tetrafluoroborate Multimedia component 21

Multimedia component 22_1-Hexanesulfonic acid sodium salt Multimedia component 22

Multimedia component 23_1-Nitroso-2-Naphthol Multimedia component 23

Multimedia component 24_2- Aminobenzothiazol Multimedia component 24

Multimedia component 25_2- Aminothiazol Multimedia component 25

Multimedia component 26_2- Mercaptopyrimidine Multimedia component 26

Multimedia component 27_2-_spl_5-Bromo-2-pyridylazo_spl_-5- diethyl aminophenol Multimedia component 27

Multimedia component 28_2_spl_4_spl_6-Tri_spl_2-pyridyl_spl_-s-triazine Multimedia component 28

Multimedia component 29_2-hydroxybiphenyl 98 _spl_ Multimedia component 29

Multimedia component 30_2-Mercaptobenzimidazole Multimedia component 30

Multimedia component 31_2-Mercaptobenzothiazole Multimedia component 31

Multimedia component 32_3-_spl_2-pyridyl_spl_-5_spl_6-diphenyl-1_spl_2_spl_4-triazine-p-p_spl_ disulfonic acid Multimedia component 32

Multimedia component 33_3_spl_3- Diamino-benzidin -tetrahydrochlorid dihydrat Multimedia component 33

Multimedia component 34_3_spl_5-Diaminobenzoic acid 98_spl_ - 27b 2_spl_3-dihydroxybenzoic acid Multimedia component 34

Multimedia component 35_4- Aminiosalicilyc acid Multimedia component 35

Multimedia component 36_4- Nitro-benzolazo 4-_spl_1-naphthol_spl_ Multimedia component 36

Multimedia component 37_4- Nitrocatechol Multimedia component 37

Multimedia component 38_4-_spl_2-pyridylazo_spl_ Resorcinol Multimedia component 38

Multimedia component 39_4-_spl_2-Thiazolylazo_spl_resorcinol Multimedia component 39

Multimedia component 40_4-Chlorophenol Multimedia component 40

Multimedia component 41_4-Chlorophenyl sulfoxide Multimedia component 41

Multimedia component 42_5- Amino-1_spl_3_spl_4-Thiadiazole-2-thiol Multimedia component 42

Multimedia component 43_5- Sulfosalicilyc acid Multimedia component 43

Multimedia component 44_5-_spl_4-Dimethylamino-benzyliden_spl_-rhodanin Multimedia component 44

Multimedia component 45_5_spl_6_spl_Diphenyl -3_spl_2-pyridyl_spl_-1_spl_2_spl_4-triazine_spl_ar_spl_-4_spl_4_spl__spl_-disulfonsaure Natrium salt Multimedia component 45

Multimedia component 46_8-Hydroxyquinoline Multimedia component 46

Multimedia component 47_a- Benzoin oxime Multimedia component 47

Multimedia component 48_Acetylacetone_spl_ 42b Cupric acetylacetone Multimedia component 48

Multimedia component 49_Ammonium hexacyanoferrate _spl_II_spl_ hydrate Multimedia component 49

Multimedia component 50_Ammonium pyrrolidinedithiocarbamate Multimedia component 50

Multimedia component 51_Antipyrin Multimedia component 51

Multimedia component 52_Barbituric acid Multimedia component 52

Multimedia component 53_Bi_spl_cyclopentadienyl_spl_titanium dichloride Multimedia component 53

Multimedia component 54_Bi_spl_cyclopentadienyl_spl_zirconium dichloride Multimedia component 54

Multimedia component 55_Bismuthiol I Multimedia component 55

Multimedia component 56_N_spl_N-Diethyl-p-phenyl-enediamine sulfate salt Multimedia component 56

Multimedia component 57_Calconcarbonsaure Multimedia component 57

Multimedia component 58_Cibracon Blue F3G-A Multimedia component 58

Multimedia component 59_Cytidine_spl_ cell culture Multimedia component 59

Multimedia component 60_di- Ammonium hydrogen citrate Multimedia component 60

Multimedia component 61_Dibenzoylmethane Multimedia component 61

Multimedia component 62_Dimethylglyoxim Multimedia component 62

Multimedia component 63_4-Methy Catechol Multimedia component 63

Multimedia component 64_Diphenylcarbazone Multimedia component 64

Multimedia component 65_Dithiooxamid Multimedia component 65

Multimedia component 66_Dithizone Multimedia component 66

Multimedia component 67_Epichlorohydron Multimedia component 67

Multimedia component 68_Fluorescein Sodium Multimedia component 68

Multimedia component 69_Gluconic acid potassium salt_spl_ sodium salt Multimedia component 69

Multimedia component 70_HEDTA Multimedia component 70

Multimedia component 71_Hippuric Acid 98_spl_ Multimedia component 71

Multimedia component 72_Hydrazine sulfate Multimedia component 72

Multimedia component 73_Mercury Ionophore I Multimedia component 73

Multimedia component 74_Michler_spl_s Ketone Multimedia component 74

Multimedia component 75_Murexid Multimedia component 75

Multimedia component 76_N- Benzoyl-N-Phenylhydroxylamine Multimedia component 76

Multimedia component 77_N_spl_N_spl_N_spl__spl_N_spl_ - Tetramethyl-1_spl_8- naphthalene diamine Multimedia component 77

Multimedia component 78_N-Hydroxysulfosuccimide sodium salt Multimedia component 78

Multimedia component 79_Nicotinic Acid Multimedia component 79

Multimedia component 80_Nitroso-R-Salt Multimedia component 80

Multimedia component 81_o-dianisidin Multimedia component 81

Multimedia component 82_Orotic acid Multimedia component 82

Multimedia component 83_Oxalate Sodium Multimedia component 83

Multimedia component 84_Phenyl Acetate 99 _spl_ Multimedia component 84

Multimedia component 85_Pyridine 2_spl_6 dicarboxylid acid Multimedia component 85

Multimedia component 86_Quinaldic Acid 98 _spl_ Multimedia component 86

Multimedia component 87_Rhodizonic acid disodium salt Multimedia component 87

Multimedia component 88_Sodium cyanide Multimedia component 88

Multimedia component 89_Sodium dibenzyldithiocarbamate Multimedia component 89

Multimedia component 90_Sodium diethyldithiocarbamate trihydrate Multimedia component 90

Multimedia component 91_syn-2-Dyridinealdoxime Multimedia component 91

Multimedia component 92_Thymine Mynimin 93 _spl_ Multimedia component 92

Multimedia component 93_Titriplex II - ethylenedinitrilotetraacetic acid Multimedia component 93

Multimedia component 94_Triethylenetriamine-N_spl_N_spl_N_spl__spl_N_spl__spl__spl_N_spl__spl__spl_N_spl__spl__spl_ hexaacetic acid Multimedia component 94

Multimedia component 95_Trioctylphosphine oxide Multimedia component 95

Multimedia component 96_Xylenol Orange Sodium Salt Multimedia component 96

Multimedia component 97__spl__spl__spl__spl__spl_ -Tetraacetic acid Multimedia component 97

Multimedia component 98_-Allylthiourea Multimedia component 98

Multimedia component 99_Menthol Multimedia component 99

Multimedia component 100_Cupferron Multimedia component 100

Multimedia component 101_Thiourea Multimedia component 101

Multimedia component 102_Strach Multimedia component 102

Multimedia component 103_Toluene-3_spl_4-dithiol Multimedia component 103

Multimedia component 104_1_spl_1 carbolyldi _spl_1_spl_2_spl_4 triazole_spl_ Multimedia component 104

Multimedia component 105_L-Carnosine Multimedia component 105

Multimedia component 106_Uracil Multimedia component 106

Multimedia component 107_1_spl_8_spl_9 Anthracenetriol Multimedia component 107

Multimedia component 108_3_spl_3_spl_ Diaminobenzidine Multimedia component 108

Multimedia component 109_o-phenanthrolin Multimedia component 109

Multimedia component 110_citric acid Multimedia component 110

Multimedia component 111_Arsenazo III Multimedia component 111

Multimedia component 112_Ferrocene Multimedia component 112

Multimedia component 113 EVA blank Multimedia component 113

Multimedia component 114 Hg_CCS Multimedia component 114

Multimedia component 115 Hg_dithizone Multimedia component 115

Multimedia component 116 Hg_resorcinol _spl_PAR_spl_ Multimedia component 116

Multimedia component 117 Hg_thiourea Multimedia component 117

Multimedia component 118 Mylar blank Multimedia component 118
